# Supplementary material for: Factors influencing the delivery and uptake of early infant diagnosis of HIV services in Greater Accra, Ghana: A qualitative study
Source: PLoS One. 2021 Feb 17;16(2):e0246876. doi: 10.1371/journal.pone.0246876 (PMC7888588; doi:10.1371/journal.pone.0246876)
Supplement: S1 File — (PDF) [file pone.0246876.s001.pdf]

INTERVIEW GUIDE FOR HEALTHWORKERS

	Unique No: 001
Date	7th June
Location	Facility A
Principal Investigator	AntoinetteAnkrah

**Background Information on participants**	
Age	
Sex	Female
Current Position	Midwife
Duration	6 years

1. What do you know about PMTCT?
   A strategy to prevent pregnant HIV Positive mothers from transmitting the virus to their infants

2. Have you enrolled in any PMTCT training? (how long ago)
   I am a trainer of trainer for PMTCT

3. What is EID?
   It is another strategy to detect HIV in exposed infants. Exposed infants is like babies born to HIV mothers

4. Was EID part of the models in the training?
   Sure, it is always included

5. What specific role do you play in the provision of EID services in this facility?
   Since I am trainer of trainers I do supervision, sometimes I do the test myself

6. How are HIV exposed infants identified for testing in this facility?

Three days after delivery, they are captured at the labour ward or post natal ward, those that we miss during the 3 days, from 7-10 days we get them a post natal, 6 weeks when they come for the child welfare clinic or some too come to the ART clinic for the cotrimoxazole for the infants, there too we capture them.

The testing is from 3 to 10 days, so any day within those days when we get them we can test them and 6 weeks we test them again to differentiate between those who are breastfeeding and those doing exclusive infant commercial feeds. Those we do exclusive infant commercial feeds if they test negative at 6 weeks then they are not going to get the cotrimoxazole because the risk is minimal but those who are actually breast feeding will still get the cotrimoxazole, even when they test negative because the risk is there when they stop breastfeeding at one year, six weeks after breast feeding, we also do EID

7. When exactly are mothers expected to bring their exposed infants back to the facility for testing?

Three days after delivery, they are captured at the labour ward or post natal ward, those that we miss during the 3 days, from 7-10 days we get them a post natal, 6 weeks when they come for the child welfare clinic or some too come to the ART clinic for the cotrimoxazole for the infants, there too we capture them.

The testing is from 3 to 10 days, so any day within those days when we get them we can test them and 6 weeks we test them again to differentiate between those who are breastfeeding and those doing exclusive infant commercial feeds. Those we do exclusive infant commercial feeds if they test negative at 6 weeks then they are not going to get the cotrimoxazole because the risk is minimal but those who are actually breast feeding will still get the cotrimoxazole, even when they test negative because the risk is there when they stop breastfeeding at one year, six weeks after breast feeding, we also do EID

8. When are the specific tests performed on infants? (virological and serological tests)

From the 3 days to the 6 weeks we do the PCR and we do the antibody test to 1 ½ years

9. Why is testing not at 4 -6 weeks or 6 weeks per National and WHO guidelines?

Well, in the past we were doing the first test at 6 weeks but now we do the first test a few days after birth because it has been realised over the years that the number of exposed infants tested after post-natal are smaller as compared to the number of mothers found to be HIV positive during the antenatal period. So we noticed that a lot of mothers don't come to the hospital at the end of the post-natal period so if we wait and test the children at 6 weeks then we will miss them.

10. How are the tests carried out? (use of protocols, procedures)

The mother brings the baby, she is counselled because she doesn't know what we are doing, though we have told her during antenatal that when the baby is born we will take sample, so when baby is born and we talk to mother about prevention of HIV and this being one of the strategy, the mother sits with the baby on the lap and we take the sample from either the heel, big toe or third and fourth finger. When the babies are too small, we take it from the heel and as they progress like 6 weeks there about we take from the big toe. You may find a child at 8 months, and that one you can take from the third or fourth finger because they are old After, you need to press hard at the site that you took the blood so that the blood will stop, because you are not going to plaster it or bandage it, if you plaster it, it may go and bleed somewhere else. So you have to make sure the bleeding stops in the clinic before you allow the mother to go and you need to counsel the mother on the site that was pricked, it she leaves and its bleeding she needs to return to the facility.

11. Why is it important for mothers to return to the facility after delivery to test their infants?

    It is only through testing that we can know the status of the child and take immediate action if the child is already infected. Also if the child tests negative, we will advise the mother on ways to prevent the child from being infected.

12. How long does it take for specimen collected to leave the facility to the central laboratory?

It could be four hours but it needs to be properly dried up first, so we normally take the samples on Friday, so by Monday morning it should be in the facility where they are going to do the test

13. How long does it take for specimen collected to be returned to the facility?

    Normally, it should be within 2 weeks but because we have challenges with the PCR machine, sometimes you can do a test and not get results in a year. There are some tests that we have done that we have never got results.

    In such instances you have to re-do the test, because there are three times that you will have to do the test before discharging the infants into the child welfare cline or ART clinic, so you will always get them to do the test.

14. What are the modes of delivery of results to the facility? (transport, sms, email etc)

    Through transport, we send the samples ourselves

15. What are some health system factors that impede delivery of EID services? (stock out of DBS kits, logistics, long turnaround time, limited staff, inadequate knowledge of staff)

    Mother lost to follow up – Some stop coming to the facility because whenever they come the PCR result is not ready so they get feed up.

    No shortage of DBS- Ridge Hospital is a facility that has lots of exposed infants so we have lots of cards and the way we do our work, we are a facility where babies just don't get positive results so NACP always gives us lots of cards, we rather give to other facilities

    Staff trained?

    We have trained mid wives at the labour ward, we have trained some for post-natal and child welfare clinic and at ART almost everyone at the ART clinic is trained EID, we don't mind whether you are a Person Living with HIV who has undergone training, we train people on the job and we are doing task shifting and task sharing so even my data manager can effectively take sample. When we started doing HIV training I was the

only nurse at the ART clinic so the drivers and cleaners were all taught how to do HIV testing before the NACP decided to introduce task shifting and task sharing

PCR machines?

Challenges with the return of PCR results. There is only one PCR machine in this region and it's situated at the Korle bu Hospital, so all health facilities that provide EID services send their samples collected to the reference lab. Just imagine the pressure that will be on the PCR machine. Often the machine breaks down or their there is a lot of back logs so it takes several months before we receive the results. At times in 3 months still the results is not in

Logistics- Vehicles transferring samples

I have a unique way of doing my work so my people have learnt it. If there is no vehicle we pick a taxi, taxi from here to Korle bu is 15 cedis so 30 cedis in and out. Last two weeks it was one of my staff who carried the sample with her own money, if I am around, I give them money. We were initially, given vehicles but the hospital has taken over the vehicle, we don't even see it. So with viral loads, we carry ourselves, the hospital doesn't care about ART.

16. Are there any experiences you will want to share with me with respect to EID?
    Pregnant women go through the normal ANC but we have special counselling with them at the ART so we have a pregnancy school, so we talk to them about the dos and don't and then this our partner support group thing too and we have mother support group, HIV positive mothers who have successfully breastfed and their babies have turned out negative also have a club, so they tend to talk to these mothers and we have some funding from USAID so we train midwives and private midwives in this catchment area, we have one big one in Nima, Maamobi area, Hajia Ada Amata, she was a nurse but has retired and has a private clinic now so if a pregnant positive mother finds herself there she will be referred to ridge .So this trend is what has empowered us as a clinic and the mothers who have HIV positive. This is how far we have come.

17. Are there any recommendations you will like to propose to improve delivery of services?

There should be more PCR machines allocated to this region because the problem for the low EID coverage is the long time for the result to return and nothing else. So to me I believe that if we get more PCR machines this problem can be resolved.

INTERVIEW GUIDE FOR HEALTHWORKERS

	Unique No:002
Date	
Location	Facility B
Principal Investigator	ANTIONETTE ANKRAH

**Background Information on Participants**	
Age	
Sex	FEMALE
Qualification	NURSE
Current Position	SENIOR STAFF NURSE/ A.R.T UNIT IN CHARGE
Duration	6 YEARS

1. What do you know about PMTCT?
   PMTCT in full means that the Prevention of Mother to Child Transmission of HIV and under PMTCT we aim to have every pregnant mother tested at the first trimester of pregnancy or as early as possible to know whether the mother is HIV positive or not. If the mother is negative, the mother is retested after 34 weeks, if the mother is positive, the mother is assisted to start the ARVs to reduce the viral load so that she doesn't pass the infection unto the unborn child.

2. Have you enrolled in any PMTCT training? (how long ago)
   Yes 5 years ago

3. What is EID?
   Early infant diagnostics seeks to test the baby in the early days of their lives within the first 3 to 7 days and then at 6 weeks. Initially, they were doing the EID at 6 weeks and it was change be within the first 3 to 7 days

4. Was EID part of the models in the training?
   Yes

5. What specific role do you play in the provision of EID services in this facility?
   My specific role is to counsel the mother on every visit that when your child is born with the first 3 to 7 days your child has to be tested, that is early infant diagnosis. We always counsel them that when they deliver, even when the midwives at the maternity wards forget to tell them to go and test they should also see it as a responsibility for themselves to remind the midwife

6. How are HIV exposed infants identified for testing in this facility?
   Every child born to an HIV positive mother is an exposed infant, so from the beginning an identified target

7. When exactly are mothers expected to bring their exposed infants back to the facility for testing?
   First 3 to 7 days and then at 6 weeks

8. Why is testing not at 4 -6 weeks or 6 weeks per National and WHO guidelines?
   It is a new practice that has been introduced

9. When are the specific tests performed on infants? (virological and serological tests)
   DNA PCR Test is done during the first 3 to 7 days and then at 6 weeks old
   Antibody test is done at one and half years

   How are the tests carried out? (use of protocols, procedures)
   We have what we call the DBS that is the dried blood spot or dried blood stain it's a sheet of paper which has some circles in it. And so when they send the baby to the ANC, the one who is taking the sample will either prick the heel of the baby and then put the spots of the blood in the circles that have been designated, then the person keeps the sample to be to be transported to Korle Bu

   Why is it important for mothers to return to the facility after delivery to test their infants?
   Because during counselling we teach them the three modes of transmission for a pregnant mother, that is the mother can pass it to her unborn child whiles its in the

womb, whiles delivering and after delivering, the breastfeeding so we need to let them come back to the facility to have the child tested, maybe the child got it from the womb so we can know as early as possible so that we can start treatment for the child. If the child is negative too then we can put in our preventive measures so that the child doesn't become positive

10. How long does it take for specimen collected to leave the facility to the central laboratory?

    The hospital sends some samples to Korle Bu so I know there is an arrangement between the ANC and the lab so when the lab is sending some samples or whatever they have to send on those designated days, they send the samples. I can't tell the frequency but I know there is an arrangement

    How long does it take for specimen collected to be returned to the facility?

    About 4 weeks or more depending on the condition of the machine it breaks down, there are times that mothers have done their test over and over and they still don't get their results. Sometimes it takes up to 4 or 6 months before they get their results.

11. What are the modes of delivery of results to the facility? (transport, sms, email etc) Transport, lab technician sends it

12. What are some health system factors that impede delivery of EID services? (stock out of DBS kits, logistics, long turnaround time, limited staff, inadequate knowledge of staff)

    Yes there are times where there are limited trained staff, at some time I think we had about just two staff trained at ANC so if one is on leave and one is not available, mothers will come and complain that they said, they are not there so I should go and come another time.

    The mothers are de motivated because, they will take the sample and send it to Korle Bu when they come for review, paediatricians are on them where are your results they go back its not ready. So they don't want to be doing it because the results are not

coming. Mostly a lot of them come back after sometime when they notice that baby is not well.

The machine is another problem, the PCR machine is a great problem, sometimes we need to know the status of the infant as early as possible, but we are not getting the results.

13. Are there any experiences you will want to share with me with respect to EID?
    I don’t have personal experiences but it’s just the discomfort that the mother go through, the mothers are willing test their infants but we tell them It is a requirement, and if the child gets infected in your womb how will we know if we don’t test and in the early days of their lives (baby) we cannot use the antibody test because the results may be coming from the mother because the child will definitely have antibodies from their mother. They are willing to do it but sometimes the person to take the sample is not there and they have to come another time and then when finally the sample are taken the results are not coming and they are frustrated, they don’t know if whether indeed their children are infected or not.

14. Are there any recommendations you will like to propose to improve delivery of services?
    I think we need to increase the number of functional PCR machines I think we as staff are ready to take samples they should work on the machines.

INTERVIEW GUIDE FOR HEALTHWORKERS

	Unique No: 003
Date	
Location	Facility B
Principal Investigator	

**Background Information on participants**	
Age	
Sex	Female
Current Position	Senior Staff nurse
Duration	8 years

1. What do you know about PMTCT?

   Preventing the unborn child from contracting HIV from the HIV positive mother
2. Have you enrolled in any PMTCT training? (how long ago)

   yes, 2 or 3 years
3. What is EID?

   Testing the infant born to an HIV mother to find out if she has the virus or not
4. Was EID part of the models in the training?

   Yes
5. What specific role do you play in the provision of EID services in this facility?

   I do PMTCT for the pregnant mothers and after delivery too, they come back and we still continue with the medication on the PMTCT and at the 6 weeks we do DBS testing for the infants. (taking samples of infants)
6. How are HIV exposed infants identified for testing in this facility?
7. When exactly are mothers expected to bring their exposed infants back to the facility for testing? At 6 weeks and 1 ½ years
8. When are the specific tests performed on infants? (virological and serological tests)

   When the child is born, we do the DBS at 6 weeks and the 1 ½ we do the antibody testing using the first response tes kit
9. How are the tests carried out? (use of protocols, procedures)

When they come, we take the babies then we take the sole of their feet. We have a card purposely for them. So the card has 5 spots in which we keep the blood but then you make sure the blood doesn't get into contact with the skin of the baby. So we take 5 samples into the circle on the card, it shouldn't go outside of the circle

10. Why is it important for mothers to return to the facility after delivery to test their infants?
11. How long does it take for specimen collected to leave the facility to the central laboratory?

    Three days, after testing on Friday, we dry it for 3 days and then first thing on Monday morning, we sent it to korle bu
12. How long does it take for specimen collected to be returned to the facility? It varies. Sometimes, as far as a month because the machine breaks down and sometimes the work over load, sometimes if the test is not well done or if we don't present the blood quantity they want, it test might be rejected.
13. What are the modes of delivery of results to the facility? (transport, sms, email etc)Through transportation.
14. What are some health system factors that impede delivery of EID services? (stock out of DBS kits, logistics, long turnaround time, limited staff, inadequate knowledge of staff)

    As far as I am concerned we don't short cards, are staff are well trained, if it should be anything, it should come from Korle bu where the PCR analysis is done
15. Are there any experiences you will want to share with me with respect to EID?

    We had a client from Kumasi who was diagnosed when she was pregnant and when she was told, she never believed it. She lived with her husband who could read and write so all she could do was to tear the antenatal card so her husband could not see what was written in it so she kept the child at home. She didn't even go to the hospital to take her medicine not to even talk of the baby's so they were home and she was doing everything (breastfeeding, cleaning) and she infected the child yet again and they were still there. When they came, the baby was around 8 months but the baby looked like a 3 month old baby. I don't know what was running through her head when she did that. Probably scared of losing the husband so she wouldn't even tell him what was happening.

    Mothers are always eager to know the status of their infants

16. Are there any recommendations you will like to propose to improve delivery of services?

    A lot of education needs to be done at the antenatal clinic, we at the ART can’t do it all we can only do provide education to the exposed ones that we see here, most of them are at the Antenatal clinic. The nurses at the antenatal need to be trained because they don’t know too much they don’t make follow ups to ensure that the newly identified ones have come to seek treatment.

INTERVIEW GUIDE FOR HEALTHWORKERS

	Unique No: 004
Date	7th June, 2018
Location	Facility A
Principal Investigator	Antoinette Ankrah

**Background Information on participants**	
Age	
Sex	Female
Current Position	Model of hope and counsellor
Duration	11 years

1. What do you know about PMTCT?
   It is an intervention given to HIV positive pregnant women that helps us to prevent mothers transmitting the HIV virus to their unborn babies.

2. Have you enrolled in any PMTCT training? (how long ago)
   Yes, about a year. 2 years ago, there was another training

3. What is EID?
   It is a test that is done for exposed babies
4. Was EID part of the models in the training?
   Yes

5. What specific role do you play in the provision of EID services in this facility?
   I take samples of the exposed babies, I also do counselling on how mothers can prevent their babies from getting infected.

6. How are HIV exposed infants identified for testing in this facility?

From the initial stage, mother is being tested at the time of pregnancy and when mothers are positive that is when we keep track on them until they give birth. We also make sure that at the point of birth, the infants are given their interventions.

7. When exactly are mothers expected to bring their exposed infants back to the facility for testing?
   6 weeks

8. When are the specific tests performed on infants? (virological and serological tests)
   PCR. Th**e PCR** is able to detect very early, at an early stage if the baby is infected but the subsequent ones that is after one year and one and half are the **antibody tests** that we do.

9. How are the tests carried out? (use of protocols, procedures)
   When babies get to 6 weeks, mothers come with the baby and samples is taken from the heel of the baby. So for the tiny babies as we always term them, those ones are taken from the heel then those that are a bit weighty, we take it from the big toe. We have this card that we have to drop the blood in 5 categories. We have to make sure the blood sample drops in the inner circle. It shouldn't spill out. You should make sure we get 3 accurate ones so that we can take it to Korle Bu.

   Are people trained to do the testing?

   I was fortunate because the time that we had this program, it was my boss Auntie Mercy and two other mid wives who went for the training and unfortunately, they were all old so working on these babies, it wasn't that easy. They have to struggle with them and all that. So when they came , I think the second week, Auntie Mercy said , "you come!" so that was how I got my training so even it took a very long time before my colleagues to learn from me but these days I hardly do it because a lot of them know how to take the sample. It was even after that that I went for the formal training. So after the blood is dropped in the circle, we dry it. Usually we take our samples on Fridays so we dry in in the rack from Friday, Saturday, Sunday then Monday you pack it with the form attached. You take the particulars of the baby; the name of the baby, date of birth, sex, name of caregiver then we attach it to the sample. We bag it: there's a particular bag for it.

10. Why is it important for mothers to return to the facility after delivery to test their infants?

    That is the only way we can help the baby to survive. Because if mothers come to test the baby and he is positive, he will be put on treatment that will make him survive

11. How long does it take for specimen collected to leave the facility to the central laboratory?

    Usually we take our samples on Fridays so we dry in in the rack from Friday, Saturday, Sunday then Monday you pack it with the form attached.

    12. How long does it take for specimen collected to be returned to the facility?

    After two to three weeks if all things being equal we get back our result and that is when we get to know whether this baby was infected in the womb or not. Sometimes due to power cuts, machines not working, they need to keep samples in the fridge for some weeks and the quality of the sample is also another issue because we don't know how well it was preserved so some of the results return as invalid and you need to call back the care giver to bring the baby for another test

12. What are the modes of delivery of results to the facility? (transport, sms, email etc)

    By vehicle. The hospital car usually picks it up. Sometimes we also have challenges with the vehicle, you request for a car and it's like the car has gone somewhere director has gone for a meeting or we pick trotro and send it or auntie mercy sends it.

13. Can you explain how mothers of HIV exposed infants are informed of the return of results?

    Mothers are counselled right from the time they are tested positive and you let them know the probabilities in these things as to whether your baby will come out negative or positive. If babies come out negative, we all thank God because that one you could see that at least all the job that you did has paid off and those that come out positive, it is difficult; that one, if I don't tell you then I'm a big liar. It is very difficult because looking through the results of other children who are negative then you come across just one that is positive then it's like you whole day is messed up, You don't even know how to call this mother and explain some of these things to them. Although you

have done a lot of counselling with them but it is not that easy but we make sure we counsel them to understand that there is still hope for those kids

14. What are some health system factors that impede delivery of EID services? (stock out of DBS kits, logistics, long turnaround time, limited staff, inadequate knowledge of staff)

    We don't run out of the dry blood samples, staffs are well involved and well knowledgeable as well.

15. Are there any experiences you will want to share with me with respect to EID?

    None

16. Are there any recommendations you will like to propose to improve delivery of services?

    None

INTERVIEW GUIDE FOR HEALTHWORKERS

	Unique No: 005
Date	
Location	Facility A
Principal Investigator	Antoinette Ankrah

**Background Information on participants**	
Age	
Sex	Female
Current Position	Model of hope
Duration	9years

1. What do you know about PMTCT?
   Taking care of pregnant women who have HIV. How they will take their medicine for them not to transfer the infection to their unborn babies

2. Have you enrolled in any PMTCT training? (how long ago)
   Yes, about a year ago

3. What is EID?
   Testing infants born to HIV positive mothers at 6 weeks

4. Was EID part of the models in the training?
   YES

5. What specific role do you play in the provision of EID services in this facility?
   I do PMTCT counselling for the pregnant women and I call and remind them of coming to the clinic for their medication and to test their infants too

6. How are HIV exposed infants identified for testing in this facility?

Because the mothers are already clients here, we take them through these counselling during the first 3 months of their pregnancy, 2nd 6 months and 8th month of the pregnancy. So they come for these counselling and we tell them what to do after delivery

7. When exactly are mothers expected to bring their exposed infants back to the facility for testing?
   6 weeks and 1 ½
8. When are the specific tests performed on infants? (virological and serological tests)
   We do the PCR at 6 weeks and the antibody test at 1 ½ years

9. How are the tests carried out? (use of protocols, procedures)
   You will first take the details of the baby and the mother and then you will prick the baby and then there is this card in which you put the blood and then you dry it.

10. Why is it important for mothers to return to the facility after delivery to test their infants?
    We want to make sure the babies are not infected with HIV

11. How long does it take for specimen collected to leave the facility to the central laboratory?
    We do here on Friday so we put it down from Friday to Monday then we send it to Korle Bu
12. How long does it take for specimen collected to be returned to the facility?
    Sometimes 3 months. We are told because there is only one machine in greater accra, they have a lot of pressure thus the delay

13. What are the modes of delivery of results to the facility? (transport, sms, email etc)
    The drivers take it with nurse from this facility

14. What are some health system factors that impede delivery of EID services? (stock out of DBS kits, logistics, long turnaround time, limited staff, inadequate knowledge of staff)

With us we don’t have any issues at all, we never run out of DBS cards because we always request some before they get finished.

We also have skilled staff to take samples. So I really don’t think we have any issues in this facility. If there should be anything maybe the delays in return of PCR test. The delay is often because of the backlogs at the reference lab because we have only one PCR machine

15. Are there any experiences you will want to share with me with respect to EID?

    None

16. Are there any recommendations you will like to propose to improve delivery of services?

    I think we are doing well so I don’t have any recommendation
