# Supplementary material for: Factors influencing the delivery and uptake of early infant diagnosis of HIV services in Greater Accra, Ghana: A qualitative study
Source: PLoS One. 2021 Feb 17;16(2):e0246876. doi: 10.1371/journal.pone.0246876 (PMC7888588; doi:10.1371/journal.pone.0246876)
Supplement: S2 File — (PDF) [file pone.0246876.s002.pdf]

# INTERVIEW GUIDE FOR HIV POSITIVE MOTHERS

| | |
|---|---|
| | Unique No: 001 |
| Date | |
| Location | |
| Principal Investigator | |

| **Background Information on participants** | |
|---|---|
| Age | 23 |
| Marital Status | No |
| Occupation | Security Officer |
| Highest educational level | SHS |
| Age of last child | 1 year |

## HIV POSITIVE MOTHERS RELATED FACTORS

1. What do you know about Early Infant Diagnosis? ( explore their knowledge on importance and exact age for testing and timelines)

   EID- It is done when the child turns 6 weeks, we bring the child for her to be tested for HIV. Another test is done at age one and half.

   Importance- It is very important, when it is done and the result is positive we can put the child on treatment so that it will not affect the child

2. How did you hear about this information? (source from facility or from a colleague/ elsewhere

   From the nurses at the ART Clinic during counselling sessions, they constantly remind us to bring baby for testing after birth.

3. Have you tested your exposed infants for HIV?

   Yes, when he was six weeks when I tested him

4. How old was your infant when the test was done?

   He was 6 weeks when he was tested

5. What kind of test was done? ( can you describe procedure)
   They took blood from under his leg and they have something they put it on, after they said they are going to take it to the lab to test it and they will bring the result when it is ready, they said they will do another one too at 1 ½ years but the one and half years one is just like what they did for me

6. Do you know the results of your infant?
   Yes

7. If Yes (What was the duration for return of results?)
   I received it 4 months later, the nurses said there was a lot of other people results at Korle bu (reference lab) that they were checking that is why it took long for the results to come

8. What are some challenges faced before, during and after testing (spouse, finance, distance, health workers?)
   No challenge at all I work so money to pick car to come to the hospital for my child is not a problem at all. I was told at counselling that when the baby is born I should bring her at 6 weeks to check if she has HIV so I came. I need to come because my child's health is important I don't have any problem at all

   The result took so long, at times you wish the result will come back very fast so you can know if your child is free or not.

   Health workers? – oh the nurses here are very good to use, they treat us well. Infant they have been so helpful, when they tested me and I was positive in fact I thought that was the end of my life, but they told me once I take my drugs well I will be fine like every normal person and baby will be fine too.

9. Is your partner aware of your HIV status? If yes how supportive has he been with respect to the EID service?
   I am no longer with the father of my baby but he is not aware too

10. Apart from your partner is any other person aware of your status?
    Apart from my mother no one knows

11. Are they supportive?

    My mother, she is very helpful she encourages me all the time. Yes like even if I am not working and I don’t have money my mother can even give me money to come.

## INTERVIEW GUIDE FOR HIV POSITIVE MOTHERS

| | Unique No: 002 |
|---|---|
| Date | |
| Location | |
| Principal Investigator | |

| **Background Information on participants** | |
|---|---|
| Age | 35 |
| Marital Status | No |
| Occupation | Trader |
| Highest educational level | JHS |
| Age of last child | **1YR** |

### HIV POSITIVE MOTHERS RELATED FACTORS

1. What do you know about Early Infant Diagnosis? ( explore their knowledge on importance and exact age for testing and timelines)

   EID is done when the child is 6 weeks old. Because the mother has HIV the child to must be checked if he has the HIV too so when the child is born and he is 6 weeks we bring them to check.

   Importance- Yes it is very important to check because we need to check to make sure there is no HIV virus in their blood, if there is virus in the blood then they can give the baby medicine so he won't fall sick or die

   Exact time for testing- 6 weeks and 1 ½ years
2. How did you hear about this information? (source from facility or from a colleague/ elsewhere

   From here, when I was pregnant I was educated on it.

3. Have you tested your exposed infants for HIV?

   Yes I have

4. How old was your infant when the test was done?
   Six weeks

5. What kind of test was done? ( can you describe procedure)
   They took blood from under her leg and they put it on something and they said they were sending it to Korle bu (reference lab) so when they bring the result they will tell me

6. Do you know the results of your infant?
   Yes my baby is negative, thank God but the nurses say I am left with another one when he is one and half years so I am praying everything will be fine

7. If Yes (What was the duration for return of results?)
   It took like 3 months before the results came.

8. What are some challenges faced before, during and after testing (spouse, finance, distance?)
   **Spousal challenges-** my partner doesn't even know
   **Finance –** often I have financial challenges, even today I called the doctor that I don't have money and she sent me money before I came. I passed through a lot of financial challenges because business is not good so I don't have money and I have not told anyone, it is only God I am looking up to.
   **Distance-** transportation from where I am to this place is also a problem but I have to find a way to come because my child health is very important to me.
   **Health workers attitude** – The nurses relate very well with us, it's just that at times if we don't come to the hospital early or do something wrong they get angry but that is only to correct us so that we don't repeat that act.

9. Is your partner aware of your HIV status? If yes how supportive has he been with respect to the EID service?
   No he is not aware

10. Apart from your partner is any other person aware of your status?
    No one apart from the doctor and nurses in the hospital, me I don't talk to man about my problems it is only God

**INTERVIEW GUIDE FOR HIV POSITIVE MOTHERS**

| | Unique No:003 |
|---|---|
| Date | |
| Location | |
| Principal Investigator | |

| **Background Information on participants** | |
|---|---|
| Age | 35 |
| Marital Status | Married |
| Occupation | Banker |
| Highest educational level | Tertiary |
| Age of last child | 1 year |

**HIV POSITIVE MOTHERS RELATED FACTORS**

1. What do you know about Early Infant Diagnosis? ( explore their knowledge on importance and exact age for testing and timelines)

   What I know is that with the early infants, when you give birth, they will first of all give you a medication that you will give to the child for the first 6 weeks then after that you come back and then they put the child on Septrin so the child will be on Septrin for 1 ½ years then they will then conduct test then whatever the result will be if the child is negative then the child can be cleared off the drug and if the child is positive then the child can be put on treatment

   **Importance-** it is very important so that you will know how to take care of the child so that you will not transfer the virus to the child

   **Exact age-** the first testing is 3 days and 1 ½ years is the last one

2. How did you hear about this information? (source from facility or from a colleague/ elsewhere

   We were educated over here during PMTCT counselling sessions

3. Have you tested your exposed infants for HIV?

   Yes

How old was your infant when the test was done?

4. Yes I tested her like 3 days after birth, in fact me with my first child the test was done at 6 weeks so I thought this one too will be done same but this test was done earlier

5. What kind of test was done? ( can you describe procedure)
   PCR Test, when you come, normally they have something that has circle circle so they will just prick the baby's leg and they will try and get the blood into each circle and then later we will go and then later on the result will come for it

   Do you know the results of your infant?
   Yes

6. If Yes (What was the duration for return of results?)
   My next visit, that was like in a month when I came for my next visit

7. What are some challenges faced before, during and after testing (spouse, finance, distance?)
   There were no challenges I faced, cost of transport was not a problem, I work, I earn good money and my husband also knows already so cost of transport was never a problem
   **Distance**- why will distance be a problem, the wellbeing of the innocent child is all I want so even if I had to go to all the way to heaven to check the status I will gladly do so because I know the importance
   **Spouse**- Oh my partner is very supportive in all of this
   **Health workers attitude**- They are very helpful and they make us feel very comfortable. I ask them questions if I don't understand anything and they are always willing to assist. All the nurses and doctors here are good, God will bless them all

8. Is your partner aware of your HIV status? If yes how supportive has he been with respect to the EID service?
   Yes, very supportive

9. Apart from your partner is any other person aware of your status?
   My mom. She is very supportive, what else can a mother do if not give support to her child in such condition

## INTERVIEW GUIDE FOR HIV POSITIVE MOTHERS

| | Unique No: 004 |
|---|---|
| Date | |
| Location | |
| Principal Investigator | |

| **Background Information on participants** | |
|---|---|
| Age | 36 |
| Marital Status | married |
| Occupation | Police Officer |
| Highest educational level | Tertiary |
| Age of last child | |

### HIV POSITIVE MOTHERS RELATED FACTORS

1. What do you know about Early Infant Diagnosis? ( explore their knowledge on importance and exact age for testing and timelines)

   EID – It is a test that is done for all children born to HIV positive mothers. The test is done twice, the first is at 6 weeks and then the next is at one and half years.

   Importance - For you to be sure that the child does not carry the virus

   Exact time – 6 weeks and 1 ½ years

2. How did you hear about this information? (source from facility or from a colleague/ elsewhere

   When I was first diagnosed, through counselling

3. Have you tested your exposed infants for HIV?

   Yes

4. How old was your infant when the test was done?

   At 6 weeks

5. What kind of test was done? ( can you describe procedure)

   Blood sample was done through the leg the blood sample unto a special card and the samples was taken to Korlebu (reference lab)

   Do you know the results of your infant?

   Yes, it was negative so I am waiting to do the final test when my baby is one and half years

6. Duration for return of results?

   It took about 3 months for me to receive it

7. What are some challenges faced before, during and after testing (spouse, finance, distance?)

   Financial Challenges/Spouse/ Distance- I experienced no challenge at all. My husband is supportive but it all lies on me the mother to take care of my child so it is no work for me at all to come here all the time.

   Health worker attitude- the health workers relate with us well, I haven’t seen them treating any mother badly before. They are the ones who even encourage us to be strong and focus on our drugs so how can they turn around to treat us bad.

8. Is your partner aware of your HIV status? If yes how supportive has he been with respect to the EID service?

   Yes, he is aware and like I said earlier the responsibility lies on me to ensure my child is in a good state and that is what I am doing.

9. Apart from your partner is any other person aware of your status?

   Apart from my husband no other person knows, my mother is deceased since 2016. My husband is very supportive.

INTERVIEW GUIDE FOR HIV POSITIVE MOTHERS

| | |
|---|---|
| | Unique No: 005 |
| Date | |
| Location | |
| Principal Investigator | |

| **Background Information on participants** | |
|---|---|
| Age | 46 |
| Marital Status | married |
| Occupation | Nurse |
| Highest educational level | SHS |
| Age of last child | 1 year |

**HIV POSITIVE MOTHERS RELATED FACTORS**

1. What do you know about Early Infant Diagnosis? ( explore their knowledge on importance and exact age for testing and timelines)
   It is important because I don’t want her to get some (HIV) that is why I will bring here
   Exact time – after birth and 1 ½ months
2. How did you hear about this information? (source from facility or from a colleague/ elsewhere
   From the hospital during PMTCT training the nurses tell us about all these things and they always remind us to come back and test our baby when we give birth

3. Have you tested your exposed infants for HIV?
   Yes

4. How old was your infant when the test was done?
   I tested her some few weeks after birth

5. What kind of test was done? ( can you describe procedure)
   They pull the blood from under her leg and they dropped it on a paper

6. Do you know the results of your infant?

   Yes

7. If Yes (What was the duration for return of results?)

   It took a while I think 4 months, I was told the machine was giving them problems, I even heard some people don’t get their results after a year.

8. What are some challenges faced before, during and after testing (spouse, finance, distance?)

   Apart from the delay in return of results everything was ok.

9. Is your partner aware of your HIV status? If yes how supportive has he been with respect to the EID service?

   Yes very supportive, he has HIV too so we are encouraging each other

10. Apart from your partner is any other person aware of your status?

    My two cousins, my auntie and husband

11. Are they supportive?

    Yes very supportive and treat me well, they are not scared to come close to me, we still relate like how we use to when I didn’t have HIV
