# Supplementary material for: Factors influencing the delivery and uptake of early infant diagnosis of HIV services in Greater Accra, Ghana: A qualitative study
Source: PLoS One. 2021 Feb 17;16(2):e0246876. doi: 10.1371/journal.pone.0246876 (PMC7888588; doi:10.1371/journal.pone.0246876)
Supplement: Attachment — Submitted filename: Responses to Reviewers.docx [file pone.0246876.s004.docx]

**P.O. BOX DS 2060**

**Dansoman – Accra**

**Ghana**

Dear Editor,

**RESPONSES TO REVIEWER’S COMMENTS ON MANUSRIPT**

Kindly find below responses to Reviewer’s comments.

| **No** | **COMMENTS** | **RESPONSES** |
| --- | --- | --- |
| 1. | The section on “consequences after disclosure” needs to be rewritten to answer the primary question about barriers to EID services. Does the disclosure dissuade people from testing? If so, this needs to be supported with data. Similarly, does the “attitude of health workers” encourage earlier care seeking, or does it discourage seeking later? | The section of consequence after disclosure has been deleted since it does not direct linkage with the objectives of the study |
| 2. | Given that there are only 2 hospitals and the authors are comparing practices, consider using pseudonyms. Further, are there funding/resource differences between the hospitals? It would be good to understand better why one hospital was able to innovate more than the other. | Pseudonyms have been assigned to the two hospitals as Facility A and B.  Facility A and B have described and compared as Regional and District Hospitals |
| 3. | Is it better to discuss risk reduction rather than prevention of transmission where possible | This comment is well noted. |
| 4. | Is the timing of the 6 week visit aligned with a vaccine visit? | Yes, the timing of 6 weeks is aligned to a vaccine visit, however, this study did not assess any linkage between EID service and uptake of immunisation. |
| 5. | Line 68: please define “HIV advancement” | This has been changed for clarity. |
| 6. | Not only are only 54% of exposed infants tested, but an even smaller percent receive results | This comment has been incorporated into the paper |
| 7. | Line 96: what is the age range of this prevalence? | The age range of this prevalence is 15- 49yrs |
| 8. | What is “Models of hope”? (text and tables) | Models of Hope are Persons Living with HIV who counsel newly diagnosed HIV mothers at the ART centres |
| 9. | Can you please format the table to be smaller (should fit on less than one page)? | The table has been formatted accordingly |
| 10. | If the participants were only those who were enrolled in PMTCT, there may be bias because those who are disengaged from care or did not seek care are not included. Please be sure to include this in the limitation. | We agree with this comment and have included it in the limitation. |
| 11. | If the sample size was determined by evaluating data saturation, then was data analysis conducted simultaneous to collection, or was another process like interim debriefings used? | Saturation point was reached with periodic debriefing among the research team |
| 12. | 15-20 min is very short for an interview. Can the authors explain why so short, and perhaps include this in the limitations. | Since the interviews were conducted during working hours, it was structured for a minimum of 15-20 mins in order for minimum interruption of work and time. This has been included to the limitations. |
| 13. | Line 130 please list the languages | Languages have been included as Twi and Fante |
| 14. | Lines 224-226 should be moved to the discussion | This has been moved to the discussion section |
| 15. | Line 244: was the “policy” on testing at birth something decided by each facility, or something happening commonly across the region/country? | Though birth testing is not a national policy, it is a common practice in both facilities utilised for the study. |
| 16. | WHO has recommended that official birth testing programs only begin once a 6 week program has been well established; are there concerns about the impact of birth testing on the 6 week program | This study did not assess this component. |
| 17. | Some studies on birth testing should be cited: for example: o <https://pubmed.ncbi.nlm.nih.gov/27540110> o <https://pubmed.ncbi.nlm.nih.gov/32183751> o <https://pubmed.ncbi.nlm.nih.gov/32329186> o <https://pubmed.ncbi.nlm.nih.gov/32520911> | Some of the suggested studies on birth testing have been included to the study |
| 18. | Line 284: instead of “the study” please say who pointed out the barrier | Changes have been effected accordingly |
| 19. | Line 308: which clinical symptoms are recognized by families? And at what age of infants? | Though this was mentioned by the health workers, the details of the clinical symptoms was not enquired. |
| 20. | Line 345: this is unclear if there is a cost or not. Similarly, in line 474, was the only cost about transport or are there other costs? | Though the provision of EID services is at no cost, other indirect cost such as transportation to the Facility was discussed. |
| 21. | Line 351-353: this could be clearer: is the barrier only about the importance of testing, regardless of finance? This seems to contradict the previous. | The wording has been improved to remove the seeming contradiction. |
| 22. | Line 390: the information about cleaners and driver is new information, so should first be in the results before the discussion. | Information about cleaners and drivers have been included in the result section as proposed. |
| 23. | Line 391: what similar findings? From where? | We have mentioned the country from where a similar study on task shifting was reported |
| 24. | Line 413: the use of the word “average” is too quantifiable for this paper. Better to say something like “many people reported” | Changes have been effected accordingly |
| 25. | Line 452: presenting with clinical symptoms may be too late; it would be helpful to understand more about the severity of symptoms and age of presentation | This comment is well noted. |
| 26. | Line 457: how do women manage testing and treatment when they have not disclosed? | The study did not assess this component. |
| 27. | Like 460: what about other family members, for example grandparents? | We note this comment, however, grandparents were not mentioned by the respondents |
| 28. | Line 482: may want to cite additional literature about “Respectful care” | Literature about Respectful care cited |
| 29. | It’s a nice finding that the staff have been able to be creative; perhaps this could be further emphasized in the discussion. | The creativity of the Staff have been further discussed in the relevant sections. |
| 30. | Please be careful when citing a rate – not every prevalence is a rate | This comment is well noted. |
| 31. | Citation #32 should be replaced with a peer review article, for example: <https://linkinghub.elsevier.com/retrieve/pii/S2352-3018(19)30033-5> | Citation has been revised. |
| 32. | While this is not a quantitative study, it is helpful to include a bit more about when the respondents fully agreed vs where there was more disagreement | This comment is well noted. |
| 33. | The paper requires some grammar editing; there are many sentences which should be more clearly written for a scientific publication | This paper has been thoroughly reviewed |
|  | COMMENTS FROM REVIEWER 1 |  |
|  | INTRODUCTION |  |
| 1. | The data on lowering the risk of MTCT with pre-natal ART is not clearly connected to the central topic of the paper: ensuring EID after birth. The statistics on MTCT in the abstract and the intro get confusing, and I would recommend establishing a stronger focus on EID right at the beginning. | This comment is well noted. EID has been introduced right from the onset of the paper. |
| 2. | The intro could be strengthened with some review of the literature from other sub-Saharan African countries. Much of this is reviewed in the discussion. I would recommend moving some of the literature to the intro that helps establish the goal of the paper and lays out what is already known on health care and maternal factors and why the context of Ghana might be different and in need of further study. | This comment is well noted, changes have been effected accordingly |
| 3. | The final sentence in the intro states the goal of the study. I am wondering about the word “use” as it does not seem as if the authors are looking at EID use only, but also functionality of services. Indeed, it is unclear how many of the themes in the results relate to use (e.g. Do you have evidence that the availability of logistics and supplies is affecting use?) I would carefully reword this objective sentence and ensure that all results clearly relate to the stated objective. | This comment is well noted, we have modified  the objective of the study to focus on the factors that facilitate or hinder the delivery and uptake of EID of HIV services. Based on this revision, we have consequently reworded the title of the study. |
| 4. | There is also an important distinction between initiating EID and completing the full EID cascade. Please describe the EID cascade according to Ghanaian guidelines and clarify which outcome (initiation or completion) relates to the literature you review and the objective of your study. | This comment is noted. The EID cascade based on Ghanaian guidelines, which we believe conforms to others includes;   1. Identification of exposed infant 2. EID Test 3. Return of PCR Results to Health Facility and Mother 4. If Infant is Positive – Link to Care /Initiation of ART 5. If Infant is Negative (not breastfeeding) – Reassure Family 6. If Infant is Negative (breastfeeding) – Plan rapid weaning at 6 -12 months 7. Carry out Anti-body test when infant is one and half years.   The obvious outcome is therefore completion of the cascade. |
|  | METHODS |  |
| 5. | There are many essential details of the methods missing. I strongly recommend the authors reference the COREQ checklist in their reporting and include the checklist in their resubmission. <http://cdn.elsevier.com/promis_misc/ISSM_COREQ_Checklist.pdf>. Importantly, the authors need to be more about what qualitative approach was used (Was there a theoretical framework? Did the authors intend use grounded theory or to build a theory?) | The COREQ Checklist has been used in reporting and a copy of the checklist has been the resubmission |
| 6. | The authors state they used purposive sampling, but not the criteria that guided the sampling. How was it purposive? Was there a specific type of purposive sampling you employed (e.g. maximum diversity, criterion, etc.) What sort of representation were you aiming to achieve? Was there other formal eligibility criteria (e.g. age). | Purposive sampling and Criterion sampling were utilised in the studies. This has been elaborated in the methods section |
|  | RESULTS |  |
| 7. | I recommend reworking the results section once the objective and theoretical framework are clarified. Each sub-section of the results needs to clearly feed into the central objective. Right now, it if unclear how certain sections work into the goal of assessing facilitators and barriers of EID use. For example, “Lost to follow-up” seems more like a consequence, not a barrier/facilitator; “Disclosure of HIV status” is not clearly connected to EID in the data presented | This comment is noted. Amendments have been made to the result section to reflect the central objectives of the study. |
| 8. | I’m unsure if the quote in “Denial of HIV Status” is saying that this is a barrier or a facilitator of EID. | The write up on Denial of HIV status has been revised for clarity. The quote however was maintained |
| 9. | I would recommend if the “Adequacy of skilled staff” section remains to clarify that you are assessing perceptions of adequacy of skilled staff, not actually using any formal criteria. | This comment is well noted |
| 10. | How does the turnaround time relate to the steps in the EID cascade? The months of waiting referenced- was for a final result (and completion of the cascade) or for the result of the more recent test? There is a big difference here. | Several outcomes are anticipated in a long waiting time for mothers. These mothers may either drop out and not complete the EID process or continue to wait for the return of the results. |
|  | DISCUSSION |  |
| 11. | Please include a Limitations section | A limitation section has been included |
| 12. | I am curious why the authors chose the subheading for the Discussion as they do not correspond to the stated goals of the study nor the organization of the results section. | The subheadings of the Discussion section have been revised to correspond with that of the Results section |
| 13. | There are often some results stated in the Discussion that were not stated in the Results section. Please ensure the Discussion interprets results already reported and does not bring in new findings. | All information reported in the Results section have now been incorporated in the Discussion section |
| 14. | Consider reviewing the following recent literature that gives more depth into: o System-level interventions for EID ♣ Finocchario-Kessler, S., Gautney, B., Cheng, A., Wexler, C., Maloba, M., Nazir, N., Khamadi, S., Lwembe, R., Brown, M., Odeny, T.A., Dariotis, J.K., Sandbulte, M., Mabachi, N., Goggin, K., 2018. Evaluation of the HIV Infant Tracking System (HITSystem) to optimise quality and efficiency of early infant diagnosis: a cluster-randomised trial in Kenya. Lancet HIV. <https://doi.org/10.1016/S2352-3018(18)30245-5> ♣ Schmitz, K., Basera, T.J., Egbujie, B., Mistri, P., Naidoo, N., Mapanga, W., Goudge, J., Mbule, M., Burtt, F., Scheepers, E., Igumbor, J., 2019. Impact of lay health worker programmes on the health outcomes of mother-child pairs of HIV exposed children in Africa: A scoping review. PLoS One. <https://doi.org/10.1371/journal.pone.0211439> o Complexities of the EID Casade ♣ Mofenson, L. M., Cohn, J., & Sacks, E. (2020). Challenges in the Early Infant HIV Diagnosis and Treatment Cascade. JAIDS Journal of Acquired Immune Deficiency Syndromes, 84, S1-S4. o Motherhood identify and EID completion ♣ Hurley, E. A., Odeny, B., Wexler, C., Brown, M., MacKenzie, A., Goggin, K., ... & Finocchario-Kessler, S. (2020). “It was my obligation as mother”: 18-Month completion of Early Infant Diagnosis as identity control for mothers living with HIV in Kenya. Social Science & Medicine, 112866. | Comment well noted. |

Thank you

**Ms. Antoinette Kailey Ankrah**

(Lead Author & Corresponding Author)
