# Supplementary material for: Factors influencing the delivery and uptake of early infant diagnosis of HIV services in Greater Accra, Ghana: A qualitative study
Source: PLoS One. 2021 Feb 17;16(2):e0246876. doi: 10.1371/journal.pone.0246876 (PMC7888588; doi:10.1371/journal.pone.0246876)
Supplement: Attachment — Submitted filename: Response to Reviewers.docx [file pone.0246876.s005.docx]

**P.O. BOX DS 2060**

**Dansoman – Accra**

**Ghana**

Dear Editor,

**RESPONSES TO REVIEWER’S COMMENTS ON MANUSRIPT**

Kindly find below responses to Reviewer’s comments.

| **No** | **COMMENTS** | **RESPONSES** |
| --- | --- | --- |
| 1. | It would be wonderful if the new objective was reflected in the abstract. | This comment is well noted. The abstract has been revised to include the objectives of the study. |
| 2**.** | Second paragraph of the discussion, it is not clear to me if you are suggesting the WHO revise it’s guidelines? I would word this carefully, as your data does not directly suggest guidelines should be revised, but that people are practicing earlier testing in other settings and calling into question the guidelines. | This comment is well noted. The statement on WHO revising its guidelines has been deleted to avoid ambiguity. |
| 3. | Please reflect on the generalizability of the findings. You allude to limitations of generalizability in the mothers you sampled. Was there anything about the health care workers or sites you collected data from that allows or doesn’t allow you to generalize results to other settings/populations? Expand on any limits to generalizability in the limitations section. | Like HIV positive mothers, health workers were purposively selected for the study. These health workers are directly involved in the delivery of EID services thus it will not be possible to generalise findings to the population general health work force in Ghana.  This has been included to the study limitations. |
| 4. | The discussion is much improved, but still lacks some key take-aways in terms of public health recommendations. Some of the literature I suggested in my previous comment #14 on system-level interventions might be worth reflecting on, or any other comments the authors want to make about what their findings mean for developing better EID systems. | This comment is well noted and revisions have been made accordingly  These revisions can be seen on;  Line 439 – 442  Line 452 – 454  Line 462 – 463  Line 471 – 472  Line 496 – 500  Line 511 - 515 |
| 5. | The intro has been much improved, especially since the objective has now been widened to include “facilitators and barriers”. However, the authors need to point out the gaps in the literature that their study sought to address. If all of this is known about facilitators and barriers to EID, why conduct the study? It may be that simply no study like this has been conducted in the Ghanaian context, in which case, the authors should point out. | This comment is well noted. Changes have been effected in the introduction accordingly. |
| 6. | When I asked for clarification of the outcome, I meant the health behavior outcome of interest in this study. I see you said “the obvious outcome is therefore the completion of the cascade” but it does seem like most of the research inquiry is focused on general uptake of EID (beginning the process). I think that would be important to clarify that you were interested in facilitators and barriers both to initiating EID and to completing it once started. Adding details of the EID cascade into the text would be helpful too- the end of the first paragraph of the introduction is currently misleading in making it seem like the virologic test within 4-6 weeks is all that encompasses EID. | This comment is noted. The introduction has been revised to include the EID cascade to clarify that Authors are interested in facilitators and barrier both to initiating EID and completion once started. |
| 7. | I don’t see the checklist but perhaps the editor does. In any case, it does look like the authors incorporated much of these criteria. Thank you. | The checklist was included during the earlier submission. We have however included it to the attachments. |
| 8. | I appreciate the authors saying they used “criterion sampling” but it would be beneficial to understand what the criterion was they were sampling on (mothers with certain characteristics, experiences…?) Saying you used criterion sampling is good, but you need to go a step further and elaborate on the criteria applied. You also mention “eligibility criteria” so should define that somewhere as well. | The criteria utilised for the selection of HIV positive mothers and health workers has been included. |
| 9. | The section looks better, but the quote is still a little difficult to comprehend (“so they seeking treatment…”). Can the syntax be improved? (perhaps “so [the idea of] seeking treatment…”) | The quote has been revised for clarity. |

Thank you

**Ms. Antoinette Kailey Ankrah**

(Lead Author & Corresponding Author)
